# Crocin Improves the Quality of Cryopreserved Goat Semen in Different Breeds

**DOI:** 10.3390/ani10061101

**Published:** 2020-06-26

**Authors:** Valentina Longobardi, Gianluigi Zullo, Alessio Cotticelli, Angela Salzano, Giuseppe Albero, Luigi Navas, Domenico Rufrano, Salvatore Claps, Gianluca Neglia

**Affiliations:** 1 Department of Veterinary Medicine and Animal Production, Federico II University, 80137 Naples, Italy; longobardivalentina@gmail.com (V.L.); alessio.cotticelli@unina.it (A.C.); angela.salzano@unina.it (A.S.); giuseppe.albero@unina.it (G.A.); neglia@unina.it (G.N.); 2 Department of Precision Medicine, University of Campania Luigi Vanvitelli, 80138 Naples, Italy; 3 Italian Buffalo Breeders Association, V. Petrarca 42/44, 81100 Caserta, Italy; zullomix@hotmail.com; 4 Council for Agricultural Research and Agricultural Economy Analysis-Research Centre for Animal Production and Aquaculture, S.S.7 Via Appia, 85051 Bella Muro, Italy; drufrano@tiscali.it (D.R.); salvatore.claps@crea.gov.it (S.C.)

**Keywords:** goat, sperm, oxidative stress, cryopreservation, crocin

## Abstract

**Simple Summary:**

In goat breeding, artificial insemination has a strong economic impact: It provides genetic improvement in intensive production systems and guarantees breed preservation in extensive systems. Sperm freezability is affected by several factors, such as breed species and freezing procedures. In addition, in goats, the seminal plasma enzyme phospholipase A reacts with egg yolk (component of semen extender), compromising sperm viability. Thus, seminal plasma is removed before freezing. This removal causes a decreasing level of antioxidants that prevent formation of toxic lipid peroxides with deleterious effects on fertility. Crocin is a water-soluble carotenoid that acts as an antioxidant, protecting cells and tissues against oxidation. Therefore, the aim of the present study was to assess the effect of crocin in the semen extender before cryopreservation. Five different goat breeds (Garganica, Jonica, Maltese Mediterranean Red and Saanen) were chosen to evaluate sperm qualitative characteristics, such as post-thawing sperm motility, viability, morphology and membrane integrity, as well as DNA fragmentation and intracellular ROS levels. The results highlighted that crocin supplementation in the extender decreased oxidative stress and improved sperm motility and the DNA integrity of frozen-thawed sperm in different breeds.

**Abstract:**

The effect of crocin in the semen extender before cryopreservation was evaluated on sperm parameters of 20 bucks of five different breeds: Garganica (GA), Jonica (JO), Maltese (MA), Mediterranean Red (MR) and Saanen (SA). Semen samples were centrifuged, to remove seminal plasma, divided in two aliquots and diluted with Tris-egg-yolk-based extender, containing 0 (control group) and 1 mM crocin. Crocin concentration was established after a preliminary dose trial. On fresh and frozen-thawed sperm, motility, viability, morphology, membrane integrity, DNA fragmentation and ROS levels were evaluated. The freezing process led to a decrease (*p* < 0.05) in all the sperm parameters recorded, confirming the deleterious effect of cryopreservation on goat semen. The most interesting result regarding the inclusion of crocin in the extender before cryopreservation was as follows: Crocin significantly improved (*p* < 0.05) sperm motility in all breeds, except for Mediterranean Red, compared to the control group. Furthermore, 1 mM crocin reduced percentage of spermatozoa with DNA fragmentation with a marked decrement (*p* < 0.05) in Garganica and Saanen, as compared to the control group. Finally, intracellular ROS decreased (*p* < 0.01) in the crocin-treated sperm of all breeds, as compared to the control. In conclusion, supplementation of 1 mM crocin in the extender decreased oxidative stress, improving sperm motility and the DNA integrity of frozen-thawed sperm in different breeds.

## 1. Introduction

Artificial insemination (AI) is the main reproductive biotechnology that can be applied in livestock. In goat breeding, it has a strong economic impact for the genetic improvement in intensive production systems [1], but it is also important in extensive systems, in order to guarantee breed preservation [2]. Despite the significant progress that has been made in this field, the diffusion of AI in goats is strongly penalized by the poor quality of frozen-thawed sperm confirmed by the huge variability in fertility rates [3]. It is already known that semen cryopreservation exerts detrimental effects on post-thaw semen motility, acrosomal status, plasma membrane and DNA integrity [4]. In addition, several reports attribute the low quality of goat sperm to different factors, such as age, breed, season and management practices [5,6,7,8].

In particular, the breed is one of the main factors that affect seminal characteristics and freezability in goats [6], due to the variability among different breeds and among individual bucks of the same breed [9]. Goat-sperm freezing is also impaired by the presence of an egg-yolk-coagulating enzyme in seminal plasma, called phospholipase A [10]. This enzyme, secreted by the bulbourethral glands, reacts with lecithin in egg yolk, a main component of semen extender, compromising sperm viability [10]. To avoid this spermicidal effect, goat semen should be centrifuged to remove seminal plasma before freezing. However, the removal of the seminal plasma may be deleterious to sperm by decreasing the level of antioxidants that act as free radical scavengers to protect sperm against oxidative stress triggers from the freezing-thawing procedures [11]. Oxidative stress stimulates the formation of toxic lipid peroxides with deleterious effects on fertility through disrupting sperm membrane integrity and inducing DNA fragmentation, mitochondrial alterations and apoptosis [12].

One of the strategies to prevent oxidative stress is the supplementation of antioxidant compounds to semen extender prior to cryopreservation. Antioxidants provide a cryoprotective effect improving post-thawing sperm motility, viability, membrane and DNA integrity in bovine, boar, buffalo, stallion and deer spermatozoa [13,14,15,16,17]. In goats, the supplementation of semen extender with several antioxidants, such as quercetin, vitamin C and resveratrol, improved frozen-thawed sperm quality [18,19,20]. Recent in vitro studies carried out on carotenoids, organic pigments produced by plants and algae, reported a protective effect of these substances on DNA fragmentation and lipid peroxidation in human [21] and bovine spermatozoa [22]. Crocin, a water-soluble carotenoid present in saffron, acts as an antioxidant by quenching free radicals and protecting cells and tissues against oxidation [23]. Crocin also exhibits a protective effect against lipid peroxidation and DNA damage caused by free radicals in deer [24] and ram spermatozoa [25]. Moreover, Sapanidou et al. [22] reported that the incubation of bovine sperm in the presence of crocin improved motility, viability, membrane integrity and blastocyst rate. To the best of our knowledge, the effects of crocin on frozen-thawed goat sperm have still not been investigated. Therefore, the aim of the present study was to assess the effect of the inclusion of crocin in the semen extender before cryopreservation on sperm qualitative characteristics and among five different goat breeds: Garganica (GA), Jonica (JO), Maltese (MA), Mediterranean Red (MR) and Saanen (SA). In particular, post-thawing sperm motility, viability, morphology and membrane integrity, as well as DNA fragmentation and intracellular ROS levels, were evaluated.

## 2. Materials and Methods

The reagents were bought from Merck/Sigma-Aldrich (Milano, Italy), if not specified.

### 2.1. Farm, Animals and Management

The trial was carried out between October and December 2019, in the experimental farm of the Council for Agricultural Research and Analysis of Agricultural Economy Analysis, Research Unit of Extensive Animal Production (CRA-ZOE, Potenza, Italy), on 20 bucks of five different breeds (GA, JO, MA, MR and SA). In particular, 4 bucks/breed (2–4 years age) were selected. In addition, a preliminary dose-response trial was performed on September 2019 on three bucks (4 ejaculates/buck of the same farm, to assess the ideal concentration of crocin to use during the experimental study on goat semen. Only bucks of proven fertility, according to the previous mounting season, were selected. The animals of each breed were maintained under the same management conditions, in paddocks that allowed 5 m^2^/head, and they received mixed hay ad libitum and a commercial concentrate supplementation (chemical composition: 88.20% of dry matter, 21.70% of crude protein, 23.00% of neutral detergent fiber and 1.77 Mcal kg^−1^ of net energy of lactation), according to their requirements, considering the mean body weight for each breed, following NRC recommendations [26]. Grazing was not allowed for all bucks from 15 days before the start of the trial. Experimental procedures and animal-care conditions followed the recommendations of the European Union directive 86/609/EEC. Furthermore, all procedures were approved by the OPBA—Ethics Committee for Animal Welfare of the University of Naples Federico II (Code: PG/2019/0028161).

### 2.2. Semen Collection

Semen collection was carried out by using the procedure described by Garde et al. [27]. Briefly, before semen collection, the rectum was cleaned of feces, and the preputial area was shaved and washed with physiological saline. Bucks were anesthetized by xylazine 2% (0.2 mg/kg LW Rompun^®^ 2% i.m.; Bayer, Milan, Italy), and semen collection was performed by a Bailey™ electroejaculator with an intermittent electrical stimulation. In particular, a consecutive series of 5 s pulses, each separated by a 5 s break, was performed three times. Ejaculates with urine contamination were rejected.

### 2.3. Assessment of Sperm Motility

Sperm motility was examined by phase contrast microscopy (Nikon E200) at 40× magnification on a glass slide overlaid with a coverslip and maintained on a temperature-controlled stage, at 37 °C. The percentage of motile spermatozoa was subjectively determined by analyzing five different microscopic fields for each semen sample [15].

### 2.4. Assessment of Sperm Viability and Morphology by Trypan Blue/Giemsa Technique

This method has been used as a routine staining in order to evaluate sperm viability and morphology [28]. Briefly, one drop of semen (5 μL) was placed on a glass slide and mixed with one drop (5 μL) of 0.27% Trypan blue; the mixture was spread, fixed for 2 min in paraformaldehyde solution 2% in PBS and stained with 7.5% Giemsa overnight. Sperm cells were observed under a light microscope at 40× magnification (Nikon E200) and differentiated for viability as acrosome intact live (AIL), acrosome intact dead (AID), acrosome-lost live (ALL) and acrosome-lost dead (ALD) and for morphology as normal (which includes abnormalities not reported to affect conception rates) and abnormal (which includes proximal droplets, head, mid-piece and tail defects) [29]. Two smears were analyzed for each ejaculate in fresh and in frozen-thawed samples, and the percentage of AIL sperm was considered as sperm viability.

### 2.5. Assessment of Sperm Membrane Integrity

On freshly ejaculated and cryopreserved spermatozoa after thawing, sperm membrane integrity was assessed by the hypoosmotic swelling (HOS) test, as described by Correa and Zavos [30]. The test was performed by mixing 50 µL of sperm sample with 0.50 mL of hypo-osmotic medium (0.73 g sodium citrate and 1.35 g fructose in 100 mL of distilled water, 150 mOsm) and incubating at 37 °C for 45 min. A 0.2 mL aliquot of the mixture was placed on a microscope slide warmed to 37 °C, covered with a 22 mm × 22 mm coverslip, and a minimum of 200 spermatozoa were examined, using phase contrast microscopy at 40× magnification (Nikon E200). The number of spermatozoa positive to HOS test (HOS+, having curled tails) was recorded.

### 2.6. Evaluation of DNA Fragmentation by Tunel Assay

Tunel assay was performed with the In Situ Cell Death Detection, Fluorescein Kit (In, Roche, Indianapolis, IN, USA), according to manufacturer’s instructions. Fresh and frozen-thawed sperm were fixed with 4% (*w*/*v*) paraformaldehyde in phosphate-buffered saline (PBS) for 30 min at room temperature. After fixation, the samples were centrifuged at 300× *g* for 10 min and resuspended in a permeability-enhancing solution containing 0.1% Triton X-100 in 0.1% sodium citrate for 10 min. Then the cells were washed twice in PBS–PVP and incubated in Tunel reaction mixture for 1 h at 37 °C, in a dark and humidified atmosphere. For a positive control, slides were treated with RNase-free DNase I, at room temperature, for 10 min, before incubation with the Tunel reagent. For a negative control, slides were incubated with all the components of the labeling solution, except for the terminal deoxynucleotidyl transferase enzyme. After one hour, slides were washed in PBS–PVP labeled with 1 mg/mL Hoechst 33342, for 30 min, at room temperature; they were then washed again in PBS–PVP and dropped (20 µL) on a glass slide in glycerol and overlaid with a coverslip. A minimum of 200 spermatozoa were examined in each sample by using a fluorescent microscope (Eclipse E-600; Nikon, Japan), under ultraviolet light, with excitation DAPI (460 nm for blue fluorescence) and FITC (520 nm for green fluorescence) filters. Digital images were acquired by using NIS-Elements-F software and a high-resolution color digital camera (Digital Sight DS-Fi 1C; Nikon, Japan), and the numbers of total (blue) and Tunel -positive (Tunel+, green) nuclei were registered.

### 2.7. Evaluation of ROS Levels

ROS levels were measured by spectrofluorometric analysis of Dihydroethidium (DHE), as previously described [31]. This probe is a cell-permeable compound oxidized by superoxide anion (O_2_^−^) to ethidium bromide that binds to DNA and emits red fluorescence. DHE (2 µM) was added to frozen-thawed sperm samples and incubated in the dark, at room temperature, for 20 min. Absorbance was monitored at 570 nm, using a plate reader (GloMax^®^-Multi Detection System-Promega, Milano). ROS levels were evaluated as arbitrary units of fluorescent signal.

### 2.8. Experimental Design

In the preliminary dose-response trial (Experiment 1), semen samples from three bucks (4 ejaculates/buck) were collected by electroejaculation (see below), and only the ejaculates with ≥60% motility were utilized. Immediately after sperm collection, volume (mL) measured by a conical graduated tube and concentration (10^9^/mL) estimated by a spectrophotometer (LKB Biochrom Ltd. Novaspec II, Cambridge, England) were evaluated. Each ejaculate was centrifuged 2000× *g* 5 min, to remove seminal plasma, divided in four aliquots and diluted at 37 °C with a Tris-egg-yolk-based extender, containing 0 (control group) 0.5, 1 and 2 mM of crocin. Aliquots were then packaged in 0.25 mL French straws and subjected to a combined cooling with equilibration period of 3 h at 5 °C. The straws were frozen with a four-step accelerating cooling rate protocol from 5° to −5° at 4 °C/min, −5 °C to −110 °C at 25 °C/min and −110 °C to −140 °C at 35 °C/min, in an automatic programmable biological cell freezer (IMV technology, L’Aigle, France). Then straws were plunged into liquid nitrogen (−196 °C) for storage. After thawing at 37 °C in a water bath for 30 s, sperm motility, viability, morphology, and membrane integrity, as well as DNA fragmentation, were evaluated. According to the results of the dose-response trial, a 1 mM crocin concentration was chosen.

In Experiment 2, during the breeding season, sperm (4 ejaculates/buck) were collected, evaluated and centrifuged, as specified above. Each ejaculate was divided into two aliquots and diluted at 37 °C with a Tris-egg-yolk-based extender, containing 0 (control group) and 1 mM crocin, to a final concentration of 100 × 10^6^ spermatozoa/mL. Aliquots were frozen as previously described, and on fresh (before seminal plasma separation) and frozen-thawed sperm, motility, viability, morphology, and membrane integrity, as well as DNA fragmentation and ROS levels, were evaluated. Frozen–thawed samples were evaluated within two months of storage in liquid nitrogen.

### 2.9. Statistical Analysis

Differences on sperm motility, viability, morphology, membrane integrity, DNA fragmentation and ROS production of fresh and frozen-thawed semen among breeds were analyzed by a repeated measured Linear Mixed Model ANOVA (IBM Corp. Released 2013. IBM SPSS Statistics for Windows, Version 22.0. Armonk, NY: IBM Corp), after confirmation of normality and homogeneity of variance. Breed, freezing and crocin presence were considered, respectively, as fixed factors for each analysis, with the buck as repeated effect. In particular, differences among breeds (on fresh and frozen-thawed semen, separately), between fresh and frozen-thawed semen (on the total and within each breed) and finally between the presence and absence of crocin on frozen-thawed semen (on the total and within each breed) were evaluated.

The effect of age, breed, treatment by crocin and their interactions were considered as main factors. All results are shown as mean ± standard error (SE). A value of *p* < 0.05 was considered statistically significant, whereas a tendency was considered for *p* < 0.10.

## 3. Results

The results of Experiment l are shown in Table 1. In particular, sperm motility increased significantly (*p* < 0.01) with 1 mM of crocin, compared to the other tested crocin concentrations. However, no differences were found among groups in AIL, ALL + ALD and HOS+ parameters. Interesting, a significant reduction (*p* < 0.01) of the percentage of spermatozoa with DNA fragmentation was recorded in the 1 mM group, compared to the other groups. According to these results, the 1mM crocin concentration was selected for further studies.

The analysis of fresh semen parameters was carried out to assess the starting semen quality and the differences among the selected breeds. The results regarding sperm volume, concentration, motility, viability and abnormalities are shown in Table 2. In particular, the volume of the ejaculate in MR was higher (*p* < 0.05), while SA showed the lowest (*p* < 0.05) sperm concentration and acrosome integrity compared to the other breeds. In addition, the percentage of sperm abnormalities was reduced (*p* < 0.05) in MA and MR, while sperm motility was not different among breeds (Table 2). Similarly, the percentages of HOS+ and Tunel + did not change among breeds (on average, 65.4 ± 3.4 and 10.2 ± 2.5%, respectively).

Regardless of the breeds, the freezing process led to a decrease in all sperm parameters recorded, confirming the deleterious effect of cryopreservation on goat sperm (Table 3). Furthermore, the percentage of spermatozoa with DNA fragmentation increased significantly (*p* < 0.05) in all breeds, as compared to fresh semen, except for SA, where the decrement was not significant (Table 3). In particular, among breeds, SA displays the lowest (*p* < 0.01) percentage of Tunel-positive spermatozoa, as well as the lowest percentages of motility and AIL (*p* < 0.05), compared to the other breeds. In addition, ALL + ALD was higher (*p* < 0.05) in MR, compared to all breeds; similarly, the percentage of HOS+ was higher (*p* < 0.05) in MR compared to JO (Table 3).

The most interesting results regarding the inclusion of crocin in the extender before cryopreservation of goat sperm are shown in Table 4. In particular, on frozen-thawed sperm, 1 mM of crocin significantly improved (*p* < 0.05) sperm motility in all breeds, except for MR, as compared to the 0 mM (control) group. On the contrary, no differences in sperm AIL, ALL + ALD and membrane integrity were reported between the experimental groups (Table 4). Interestingly, among breeds, crocin supplementation improved (*p* < 0.05) in SA the percentages of HOS+, although SA still showed the lowest motility (*p* < 0.05), compared to the other breeds. In addition, an increase (*p* < 0.05) in AIL was recorded in GA and MR, compared to JO and MA, while no differences were recorded for SA.

**Figure 1:**
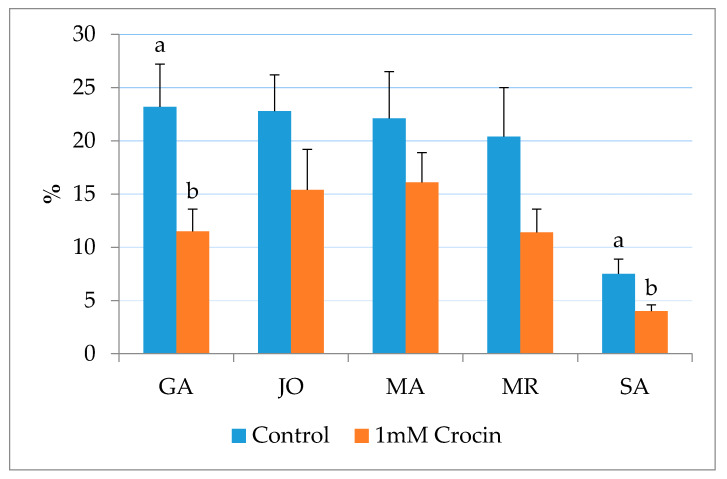
Percentages of Tunel-positive sperm (DNA fragmentation index) of frozen-thawed goat sperm in different breeds: Garganica (GA), Jonica (JO), Maltese (MA), Mediterranean Red (MR) and Saanen (SA) between 0 mM (control) and 1 mM crocin groups. ^a,b^ Bars with different letters are significantly different; *p* < 0.05.

Furthermore, 1 mM crocin supplementation caused a significant decrease (*p* < 0.01) of the percentage of spermatozoa with DNA fragmentation, compared to the control group (19.2 ± 3.6 vs. 11.7 ± 2.3, respectively), regardless of the breeds. Among breeds, a significant (*p* < 0.05) decrease in DNA fragmentation was observed in GA and SA, while no differences were detected in the crocin-treated group for JO, MA and MR, despite the marked reduction of Tunel-positive spermatozoa (Figure 1).

**Figure 2:**
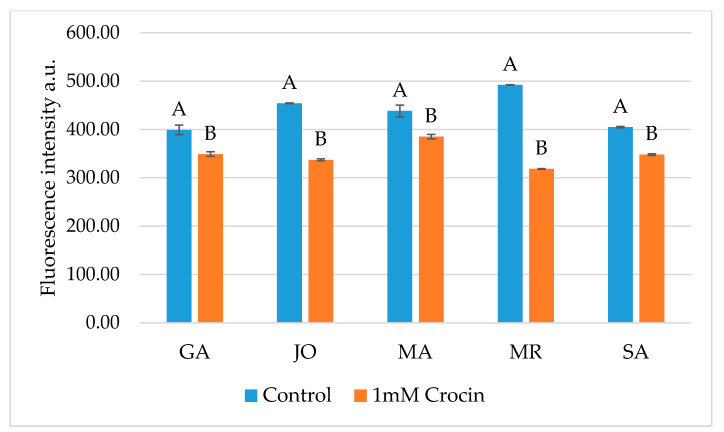
DHE fluorescence intensity of frozen-thawed goat sperm in different breeds: Garganica (GA), Jonica (JO), Maltese (MA), Mediterranean Red (MR) and Saanen (SA) between 0 mM (control) and 1 mM crocin groups. ^A,B^ Bars with different letters are significantly different; *p* < 0.01.

Finally, crocin supplementation significantly (*p* < 0.01) reduced ROS levels in each breed, compared to the control group, as shown in Figure 2. Consequently, crocin’s effect was even more evident independently of the breed (437.8 ± 4.9 vs. 347.6 ± 2.8; *p* < 0.01 in control and crocin group, respectively).

## 4. Discussion

The present trial focused on the development of strategies to improve the quality of cryopreserved goat sperm, using an experimental extender supplemented with crocin, a natural substance known for its antioxidant properties. The results of this study showed that crocin had a beneficial effect on sperm motility and DNA integrity, reducing oxidative stress of frozen-thawed goat sperm in different breeds. Semen quality variation exists from different breeds, within breed and individual bucks [32,33]. The identification of breed-specific differences in the performance of bucks should help to manage semen samples in AI programs. Breed, as well as age, nutrition, seasonality and management, influences semen goat characteristics [6]. Therefore, to limit the potential influence of these factors, the bucks involved in this study belonged to a homogenous group (similar age and diet) in the same farm and under the same management conditions throughout the experimental period. In addition, as it is known that male goats are seasonal breeders and their reproductive activities are influenced by photoperiod [34], the trial was performed in autumn, during the breeding season at our latitude.

The results on fresh semen pointed out that sperm volume and concentration differed among breeds, as well as the percentage of abnormal spermatozoa. These data are in accordance with several studies that reported significant variations in semen quality among breeds and individual bucks [31,32]. In particular, in our study, Saanen bucks showed lower sperm volume, concentration, and acrosome integrity, as well as the highest percentage of sperm abnormalities, as compared to the other breeds. Evans and Maxwell [35] reported that, on small ruminants, an ejaculate can be considered of good fertilizing quality with 15 to 20% of sperm abnormalities. Abnormally shaped or damaged sperm have been negatively correlated to fertility [36]. It is worth noting that the abnormalities recorded in this study were principally secondary abnormalities that included loose heads, droplets of cytoplasm on the tail or midpiece and bent tails. These defects occurred during the later stages of sperm development, during ejaculation or during the collection process and are usually classified as minor sperm abnormalities that may lead to subfertility [37].

Another result of this study was the evident decline in the quality of semen after cryopreservation. Previous studies reported that goat sperm does not have high adaptability to temperature changes, which may contribute to sperm sensitivity [38,39]. The semen freezing–thawing procedure is known to induce damages on sperm plasma membrane, reducing motility and acrosome integrity of goat spermatozoa [3]. In our study, regardless of breed, freezing decreased sperm motility, viability, and acrosome integrity, increasing the percentage of DNA fragmentation. These data are particularly important because DNA fragmentation negatively affects both the fertilizing capacity of spermatozoa and the subsequent embryo development in vitro [40,41].

The most interesting results emerged when crocin was added to the semen extender before freezing. The effect of crocin has been assessed on motility, vitality, membrane integrity, DNA fragmentation and ROS levels. Crocin significantly improves motility in GA, JO, MA and SA breeds, while no difference emerged for MR compared to the control group. Motility is one of the most important indicators of the potential fertilizing ability of spermatozoa [42]. Similar results were reported in cattle, in which 1 mM of crocin was the most effective in improving motility [22]. Likewise, in other papers, the beneficial effect of saffron and its bioactive components, including crocin, on motility, viability and membrane integrity in the spermatozoa of human, rat, rooster and rabbit were reported [43,44,45,46]. In this study, however, no differences were recorded for sperm viability and membrane integrity that were high, regardless of breeds and treatment. On the contrary, in a recent study in bovine, membrane integrity was improved in the presence of crocin [22]. These slight differences are likely attributable to species-specific peculiarities. Furthermore, crocin markedly reduced DNA fragmentation compared to the control group. Similarly, a decrease in the DNA fragmentation index was recorded in ram spermatozoa when the antioxidant was added to the extender before freezing [25]. Sapanidou et al. [22] reported a decrement of DNA integrity in the presence of 1 and 2 mM of crocin. It is already known that a positive correlation exists between ROS production and DNA fragmentation [39]. The cold shock caused by freezing and thawing procedures increases the susceptibility of semen to oxidative stress due to an increment in ROS production [47]. ROS have been shown to change cellular functions through the disruption of the sperm plasma membrane and damage to proteins and DNA [47]. The addition of antioxidants can reduce ROS production and preserve the integrity of sperm chromatin [48].

Accordingly, to reduced DNA fragmentation, crocin also showed a significant decrement in the production of superoxide anion, measured by DHE fluorescence intensity in all breeds. A reduction of ROS levels was also observed in bovine sperm treated with crocin [22]. In contrast, in red deer, crocin failed to decrease ROS production, while it was effective in protecting sperm exposed to exogenous oxidative stress [24]. The production of ROS is a normal physiological process, but freezing–thawing procedures induce an imbalance between ROS generation and scavenging activity that is detrimental to the sperm. It is known that antioxidants can be used to prevent the negative effect of oxidative stress due to the generation of ROS on sperm parameters in different species [13,14,15,16,17]. Crocin, in particular, is known to be a powerful scavenger of free radicals, especially superoxide anions, and stimulates glutathione synthesis, providing cells a protection from oxidative stress [49]. The exact mechanism by which crocin acts is not yet full elucidated; however, it is possible to speculate that the reduction of ROS levels observed in this trial may be related to the scavenger ability of this antioxidant to face against the overproduction of ROS induced by cryopreservation.

## 5. Conclusions

In conclusion, the results of this study demonstrated that crocin is able to reduce oxidative stress, with a beneficial effect on sperm motility and DNA integrity, hence on the quality of the frozen-thawed goat sperm in different breeds. These preliminary data represent an excellent starting point for further studies aimed to deepen knowledge on the mechanism of action of this antioxidant compound and to assess the effect of crocin on sperm fertilizing ability, in order to optimize the cryopreservation procedures and the diffusion of AI in this species.

## Figures and Tables

**Table 1:** Effect of different crocin concentrations on frozen-thawed semen qualitative characteristics. Data are expressed as mean values ± SEM.

Crocin	Motility (%)	AIL * (%)	ALL + ALD ** (%)	HOS+ *** (%)	Tunel+ **** (%)
0 mM	63.1 ± 0.2 ^A^	74.8 ± 1.8	25.2 ± 1.7	63.3 ±1.5	21.4 ± 4.1 ^A^
0.5 mM	65.0 ± 1.2 ^A^	75.2 ± 1.9	24.8 ± 1.8	62.9 ± 2.3	18.4 ± 2.7 ^A^
1 mM	73.3 ± 0.5 ^B^	75.1 ± 2.1	24.9 ± 2.0	66.3 ± 2.1	9.8 ± 1.6 ^B^
2 mM	57.5 ± 0.8 ^AC^	74.8 ± 2.0	25.2 ± 1.5	65.1 ± 1.6	22.8 ± 3.4 ^A^

^A,B,C^ Values within rows for each breed with different superscripts are different; *p* < 0.01. * AIL = acrosome intact live; ** ALL = acrosome loss live; ALD = acrosome loss dead; *** HOS+ = membrane integrity; **** Tunel + = DNA fragmentation index.

**Table 2:** Fresh semen qualitative characteristics in different breeds: Garganica (GA), Jonica (JO), Maltese (MA), Mediterranean Red (MR) and Saanen (SA). Data are expressed as mean ± SEM.

Breed	Volume (mL)	Concentration (10^9^/mL)	Motility (%)	AIL (%)	Abnormalities (%)
GA	0.7 ± 0.1 ^B^	6.0 ± 0.9	70.0 ± 3.0	83.1 ± 2.1	21.3 ± 4.5 ^a^
JO	0.9 ± 0.1 ^AB^	4.7 ± 1.0	68.0 ± 1.3	76.2 ± 3.9	19.5 ± 3.5 ^a^
MA	0.8 ± 0.1 ^b^	6.5 ± 0.6 ^a^	70.7 ± 3.6	82.0 ± 3.5	10.9 ± 2.1 ^b^
MR	1.1 ± 0.1 ^Aa^	5.8 ± 1.0	67.8 ± 5.6	85.1 ± 3.6 ^a^	12.1 ± 2.4 ^b^
SA	0.7 ± 0.1 ^B^	3.8 ± 0.9 ^b^	70.5 ± 2.9	69.7 ± 6.4 ^b^	23.0 ± 6.0 ^a^

^A,B^ Values within columns with different superscripts are different; *p* < 0.01. ^a,b^ Values within columns with different superscripts are different; *p* < 0.05.

**Table 3:** Fresh and frozen-thawed semen qualitative characteristics in different breeds: Garganica (GA), Jonica (JO), Maltese (MA), Mediterranean Red (MR) and Saanen (SA). Data are expressed as mean ± SEM.

Breed	Group	Motility (%)	AIL * (%)	ALL + ALD ** (%)	HOS+ *** (%)	Tunel+ **** (%)
GA	Fresh	70.0 ± 3.0	83.1 ± 2.1 ^A^	6.1 ± 1.7 ^A^	67.8 ± 2.4	7.8 ± 2.0 ^A^
	Frozen	65.0 ± 1.5	70.8 ± 2.9 ^B^	23.7 ± 2.0 ^B^	61.0 ± 3.3	23.2 ± 4.0 ^B^
JO	Fresh	68.0 ± 1.3	76.2 ± 3.9	4.7 ± 1.5 ^A^	61.0 ± 2.8	12.1 ± 3.1 ^a^
	Frozen	60.0 ± 3.8	70.2 ± 2.4	21.8 ± 2.4 ^B^	58.2 ± 1.9	22.8 ± 3.4 ^b^
MA	Fresh	70.7 ± 3.6	82.0 ± 3.5 ^A^	7.1 ± 2.5 ^A^	67.3 ± 3.3	9.7 ± 2.8 ^a^
	Frozen	65.7 ± 2.5	67.1 ± 3.2 ^B^	22.5 ± 2.8 ^B^	64.9 ± 3.2	22.1 ± 4.4 ^b^
MR	Fresh	67.3 ± 5.6	85.1 ± 3.6 ^a^	3.5 ± 1.0 ^A^	68.4 ± 2.1	9.9 ± 1.6 ^a^
	Frozen	66.0 ± 3.4	76.0 ± 2.1 ^b^	15.2 ± 1.6 ^B^	66.6 ± 3.6	20.4 ± 4.6 ^b^
SA	Fresh	70.5 ± 2.9 ^A^	69.7 ± 6.4	4.1 ± 1.6 ^A^	64.6 ± 4.9	7.5 ± 1.4
	Frozen	50.0 ± 6.6 ^B^	62.5 ± 3.2	26.0 ± 5.2 ^B^	61.5 ± 6.1	11.6 ± 3.1
TOTAL	Fresh	69.3 ± 3.3 ^a^	79.2. ±3.9 ^A^	5.1 ± 1.7 ^A^	65.8 ± 3.1	9.4 ± 2.2 ^A^
	Frozen	61.3 ± 3.6 ^b^	69.3 ± 2.8 ^B^	21.8 ± 2.8 ^B^	62.4 ± 3.6	20.0 ± 3.9 ^B^

^A,B^ Values within rows for each breed with different superscripts are different; *p* < 0.01. ^a,b^ Values within rows with different superscripts are different; *p* < 0.05. * AIL = acrosome intact live; ** ALL = acrosome loss live; ALD = acrosome loss dead; *** HOS+ = membrane integrity; **** Tunel+ = DNA fragmentation index.

**Table 4:** Frozen–thawed semen qualitative characteristics between 0 mM (control) and 1 mM crocin groups in different breeds: Garganica (GA), Jonica (JO), Maltese (MA), Mediterranean Red (MR) and Saanen (SA). Data are expressed as mean ± SEM.

Breed	Group	Motility (%)	AIL * (%)	ALL + ALD ** (%)	HOS+ *** (%)
GA	Control	65.0 ± 1.5 ^A^	70.8 ± 2.9	23.7 ± 2.0	61.0 ± 3.3
	1 mM crocin	74.2 ± 1.8 ^B^	78.1 ± 3.0	18.0 ± 2.7	63.2 ± 1.9
JO	Control	60.0 ± 3.8 ^A^	70.2 ± 2.4	21.8 ± 2.4	58.2 ± 1.9
	1 mM crocin	74.0 ± 2.7 ^B^	71.4 ± 1.3	23.8 ± 1.0	65.0 ± 3.9
MA	Control	65.7 ± 2.5 ^A^	67.1 ±3.2	22.5 ± 2.8	64.9 ± 3.2
	1 mM crocin	74.3 ± 2.0 ^B^	69.1 ±.2.0	24.1 ± 2.0	63.6 ± 2.4
MR	Control	66.0 ± 3.4	76.0 ± 2.1	15.2 ± 1.6	66.6 ± 3.6
	1 mM crocin	64.0 ± 1.6	76.2 ± 1.9	15.0 ± 1.1	67.6 ± 2.4
SA	Control	50.0 ± 6.6 ^a^	62.5 ± 3.2	26.0 ± 5.2	61.5 ± 6.1
	1 mM crocin	60.0 ± 5.8 ^b^	73.5 ± 6.0	21.5 ± 4.9	75.0 ± 3.5
TOTAL	Control	61.3 ± 3.6 ^A^	69.3 ± 2.8	21.8 ± 2.8	62.4 ± 3.6
	1 mM crocin	69.3 ± 2.8 ^B^	73.7 ± 2.9	20.5 ± 2.3	66.9 ± 2.8

^A,B^ Values within rows for each breed with different superscripts are different; *p* < 0.01. ^a,b^ Values within rows with different superscripts are different; *p* < 0.05. * AIL = acrosome intact live; ** ALL = acrosome loss live; ALD = acrosome loss dead; *** HOS+ = membrane integrity.

## References

[1] Leboeuf B., Restall B., Salamon S. (2000). Production and storage of goat semen for artificial insemination. Anim. Reprod. Sci..

[2] Gama L., Bressan M.C. (2011). Biotechnology applications for the sustainable management of goat genetic resources. Small Rumin. Res..

[3] Gangwar C., Kharche S.D., Kumar S., Jindal S.K. (2016). Cryopreservation of goat semen: Status and prospects. Indian J. Small Rumin..

[4] Bailey J.L., Bilodeau J.F., Cormier N. (2000). Semen cryopreservation in domestic animals: A damaging and capacitating phenomenon. J. Androl..

[5] Al-Ghalban A.M., Tabaa M.J., Kridli R.T. (2004). Factors affecting semen characteristics and scrotal circumference in Damascus bucks. Small Rumin. Res..

[6] Arrebola F., Abecia J.A. (2017). Effects of season and artificial photoperiod on semen and seminal plasma characteristics in bucks of two goat breeds maintained in a semen collection center. Vet. World.

[7] Qureshi M.S., Khan D., Mustaq A., Afridi S.S. (2013). Effect of extenders, post dilution intervals and seasons on semen quality in dairy goats. Turk. J. Vet. Anim. Sci..

[8] Hanmante A.A., Barbind R.P., Mule R.S., Baswade S.V., Andhare B.C. (2009). Effect of management system on semen quality attributes of Osmanabadi goat bucks. Indian J. Small Rumin..

[9] Abdi-Benemar H., Khalili B., Zamiri M.J., Ezazi H. (2018). Seasonal variation in seminal characteristics, testicular measurements and plasma testosterone concentration in Iranian Khalkhali bucks. J. Livest. Sci. Technol..

[10] Ritar A.J., Salamon S. (1982). Effects of seminal plasma and of its removal and of egg yolk in the diluents on the survival of fresh and frozen-thawed spermatozoa of the Angora goat. Aust. J. Biol. Sci..

[11] Ferreira V., Mello M., Fonseca C., Dias Á., Cardoso J., Silva R., Junior W. (2014). Effect of seminal plasma and egg yolk concentration on freezability of goat semen. Rev. Bras. De Zootec..

[12] Agarwal A., Majzoub A. (2017). Laboratory tests for oxidative stress. Indian J. Urol.

[13] Bucak M.N., Tuncer P.B., Sarıözkan S., Başpınar N., Taşpınar M., Coyan K., Bilgili A., Akalın P.P., Büyükleblebici S., Aydos S. (2010). Effects of antioxidants on post-thawed bovine sperm and oxidative stress parameters: Antioxidants protect DNA integrity against cryodamage. Cryobiology.

[14] Estrada E., Rodríguez-Gil J.E., Rocha L.G., Balasch S., Bonet S., Yeste M. (2014). Supplementing cryopreservation media with reduced glutathione increases fertility and prolificacy of sows inseminated with frozen-thawed boar semen. Andrology.

[15] Longobardi V., Zullo G., Salzano A., De Canditiis C., Cammarano A., De Luise L., Puzio M.V., Neglia G., Gasparrini B. (2017). Resveratrol prevents capacitation-like changes and improves in vitro fertilizing capability of buffalo frozen-thawed sperm. Theriogenology.

[16] Nouri H., Shojaeian K., Samadian F., Lee S., Kohram H., Lee J.I. (2018). Using resveratrol and Epigallocatechin-3-Gallate to improve cryopreservation of stallion spermatozoa with low quality. J. Equine Vet. Sci..

[17] Sánchez-Rubio F., Fernández-Santos M., Castro-Vázquez L., García-Álvarez O., Maroto-Morales A., Soler A.J., Martínez-Pastor F., Garde J.J. (2018). Cinnamtannin B-1, a novel antioxidant for sperm in red deer. Anim. Reprod. Sci..

[18] Daramola J.O., Adekunle E.O. (2015). Cryosurvival of goat spermatozoa in Tris-egg yolk extender supplemented with vitamin C. Arch. Zootec..

[19] Lv C., Larbi A., Wu G., Hong Q., Quan G. (2019). Improving the quality of cryopreserved goat semen with a commercial bull extender supplemented with resveratrol. Anim. Reprod. Sci..

[20] Seifi-Jamadi A., Ahmad E., Ansari M., Kohram H. (2017). Antioxidant effect of quercetin in an extender containing DMA or glycerol on freezing capacity of goat semen. Cryobiology.

[21] Ghyasvand T., Goodarzi M.T., Amiri I., Karimi J., Ghorbani M. (2015). Serum levels of lycopene, beta-carotene, and retinol and their correlation with sperm DNA damage in normospermic and infertile men. Int. J. Reprod. Biomed. (Yazd).

[22] Sapanidou V., Taitzoglou I., Tsakmakidis I., Kourtzelis I., Fletouris D., Theodoridis A., Zervos I., Tsantarliotou M. (2015). Antioxidant effect of crocin on bovine sperm quality and in vitro fertilization. Theriogenology.

[23] Rahaiee S., Moini S., Hashemi M., Shojaosadati S.A. (2015). Evaluation of antioxidant activities of bioactive compounds and various extracts obtained from saffron (Crocus sativus L.): A review. J. Food Sci. Technol..

[24] Domínguez-Rebolledo A.E., Fernández-Santos M.R., Bisbal A., Ros-Santaella J.L., Ramón M., Carmona M., Martínez-Pastor F., Garde J.J. (2010). Improving the effect of incubation and oxidative stress on thawed spermatozoa from red deer by using different antioxidant treatments. Reprod. Fertil. Dev..

[25] Mata-Campuzano M., Álvarez-Rodríguez M., Álvarez M., Tamayo-Canul J., Anel L., de Paz P., Martínez-Pastor F. (2015). Post-thawing quality and incubation resilience of cryopreserved ram spermatozoa are affected by antioxidant supplementation and choice of extender. Theriogenology.

[26] National Research Council (NRC) (2007). Nutrient Requirements of Small Ruminants: Sheep, Goats, Cervids, and New World Camelids.

[27] Garde J.J., Soler A.J., Cassinello J., Crespo C., Malo A.F., Espeso G., Gomendio M., Roldan E.M.S. (2003). Sperm cryopreservation in three species of endangered gazelles (Gazella cuvieri, G. dama mhorr, and G. dorcas neglecta). Biol. Reprod..

[28] Boccia L., Di Palo R., De Rosa A., Attanasio L., Mariotti E., Gasparrini B. (2007). Evaluation of buffalo semen by Trypan blue/Giemsa staining and related fertility in vitro. Ital. J. Anim. Sci..

[29] World Health Organization Department of Reproductive Health and Research (2010). WHO Laboratory Manual for the Examination and Processing of Human Semen.

[30] Correa J.R., Zavos P.M. (1994). The Hypoosmotic swelling test: Its employment as an assay to evaluate the functional integrity of the frozen-thawed bovine sperm membrane. Theriogenology.

[31] Nazarewicz R., Bikineyeva A., Dikalov S. (2013). Rapid and specific measurements of superoxide using fluorescence spectroscopy. J. Biomol. Screen.

[32] Karagiannidis A., Varsakeli S., Karatzas G. (2000). Characteristics and seasonal variations in the semen of Alpine, Saanen and Damascus goat bucks born and raised in Greece. Theriogenology.

[33] Sultana F., Husain S.S., Khatun A., Apu A.S., Khandoker M.A.M. (2013). Study on buck evaluation based on semen quality and fertility. Bang. J. Anim. Sci..

[34] Delgadillo J.A., Fitz-Rodríguez G., Duarte G., Véliz F.G., Carrillo E., Flores J.A., Vielma J., Hernandez H., Malpaux B. (2004). Management of photoperiod to control caprine reproduction in the subtropics. Reprod. Fertil. Dev..

[35] Evans G., Maxwell W.M.C. (1987). Salamon’s Artificial Insemination of Sheep and Goats.

[36] Saacke R.G., White J.M. (1972). Semen quality tests and their relationship to fertility. Proceedings of the 4th National Association of Animal Breeders, Technology Conference Artificial Insemination and Reproduction.

[37] Adeniji D.A., Oyeyemi M.O., Olugbemi J.B. (2010). Sperm morphological characteristic and mating behaviour of Proviron^®^ treated west African dwarf bucks with testicular degeneration. Int. J. Morphol..

[38] Barbas J., Mascarenhas R. (2009). Cryopreservation of domestic animal sperm cells. Cell Tissue Bank..

[39] Dorado J., Munoz-Serrano A., Hidalgo M. (2010). The effect of cryopreservation on goat semen characteristics related to sperm freezability. Anim. Reprod. Sci..

[40] Simões R., Feitosa W.B., Siqueira A.F., Nichi M., Paula-Lopes F.F., Marques M.G., Peres M.A., Barnabe V.H., Visintin J.A., Assumpção M.E. (2013). Influence of bovine sperm DNA fragmentation and oxidative stress on early embryo in vitro development outcome. Reproduction.

[41] Üstüner B., Nur Z., Alçay S., Toker M.B., Sağirkaya H., Soylu M.K. (2015). Effect of freezing rate on goat sperm morphology and DNA integrity. Turk. J. Vet. Anim. Sci..

[42] Vijayaraghavan S. (2003). Sperm Motility: Patterns and Regulation. Introduction to Mammalian Reproduction.

[43] Abdullaev F.I., Caballero-Ortega H., Riveron-Nigrete L., Pereda-miranda R., Rivera- Luna R., Manuel Hernandez J., Perez-Lopez I., Espinosa-Aguirre J.J. (2002). In vitro evaluation of chemopreventive potential of saffron. Rev. Invest. Clin..

[44] Mardani M., Vaez A., Razavi S. (2014). Effect of saffron on rat sperm chromatin integrity. Int. J. Reprod. Biomed..

[45] Mehdipour M., Kia D.H., Najafi A. (2019). Effect of crocin and naringenin supplementation in cryopreservation medium on post-thawed rooster sperm quality and expression of apoptosis associated genes. BioRxiv.

[46] Tsantarliotou M.P., Poutahidis T., Markala D., Kazakos G., Sapanidou V., Lavrentiadou S., Zervos I., Taitzoglou I., Sinakos Z. (2013). Crocetin administration ameliorates endotoxin-induced disseminated intravascular coagulation in rabbits. Blood Coagul. Fibrinolysis.

[47] Martorana K., Klooster K., Meyers S. (2014). Suprazero cooling rate, rather than freezing rate, determines post thaw quality of rhesus macaque sperm. Theriogenology.

[48] Gualtieri R., Barbato V., Fiorentino I., Braun S., Rizos D., Longobardi S., Talevi R. (2014). Treatment with zinc, d-aspartate, and co-enzyme Q10 protects bull sperm against damage and improves their ability to support embryo development. Theriogenology.

[49] Mokhber Maleki E., Eimani H., Bigdeli M.R., Golkar Narenji A., Abedi R. (2016). Effects of crocin supplementation during in vitro maturation of mouse oocytes on glutathione synthesis and cytoplasmic maturation. Int. J. Fertil. Steril..

